# Combining SFCscore with Random Forests leads to improved affinity prediction for protein-ligand complexes

**DOI:** 10.1186/1758-2946-5-S1-P27

**Published:** 2013-03-22

**Authors:** D Zilian, CA Sotriffer

**Affiliations:** 1 Institute of Pharmacy and Food Chemistry, University of Wuerzburg, Am Hubland, 97074 Wuerzburg, Germany

## 

SFCscore is a collection of emprirical scoring functions derived from a set of over 60 descriptors for protein-ligand complexes of known structure [1]. By the time of their derivation, SFCscore functions were the best-performing scoring functions tested on large heterogeneous data sets, but the overall correlation was still not within the desired range. Similarly, despite the ever increasing amount of structure and affinity data, the general advancements in the development of empirical scoring functions have been rather moderate over the past years. However, more recently, Ballester and Mitchell [2] published a function that outperformed current state-of-the-art scoring functions when tested against the PDBbind benchmark set [3]. This function uses relatively simple atom contact counts as descriptors and is derived by the Random Forest algorithm. Here, we present a study in which we used Random Forests to derive a new function ("SFCscoreRF") based on the SFCscore descriptors as input data. Although this is not a fully non-parametric approach, the descriptors are supposed to capture more accurately the physically relevant interactions. We tested the new function against the PDBbind benchmark set and the CSAR-NRC HiQ 2010 set [4] and, in addition, performed the Leave-Cluster-Out validation as proposed by Kramer and Gedeck for the PDBbind set [5]. The results suggest that the new function significantly improves the predictive power of SFCscore, as it increases the correlation between predicted and experimentally determined affinities for the PDBbind benchmark set from r^2^ = 0.41 (best previous SFCscore function) to r^2^ = 0.61 (SFCscoreRF) and for the CSAR data set from r^2^ = 0.38 to r^2^ = 0.53.
